# The Embodied Simulation of L2 Grammatical Aspect: Proficiency-Dependent Evidence from the Action-Sentence Compatibility Effect

**DOI:** 10.3390/bs15111581

**Published:** 2025-11-18

**Authors:** Chunqiao Hu, Shifa Chen, Yufeng Liu

**Affiliations:** English Department, College of Foreign Languages, Ocean University of China, Qingdao 266100, China; chenshifa99@ouc.edu.cn (S.C.); liuyufeng6126@stu.ouc.edu.cn (Y.L.)

**Keywords:** grammatical aspect, embodied simulation, action-sentence compatibility effect (ACE), second language processing, embodied cognition

## Abstract

Using the Action–Sentence Compatibility Effect (ACE) paradigm, this study investigated whether types of grammatical aspects and L2 proficiency influence embodied simulation during L2 sentence comprehension of English among Chinese learners. Participants judged the semantic plausibility of sentences in progressive or perfective aspect by performing directional actions (toward or away the body) that were either compatible or incompatible with the action direction described. Analysis of the reaction times (RTs) revealed a significant main effect of proficiency, with low-proficiency learners responding more slowly overall. Crucially, we observed a significant three-way interaction between aspect, action–sentence consistency, and proficiency. Simple effects analyses revealed a qualitative reversal: advanced learners exhibited a significant ACE only for sentences in the progressive aspect, indicating grammatically guided simulation sensitive to ongoing actions, whereas we found no ACE for perfective sentences, consistent with their focus on event completion rather than on-going action processes. In contrast, low-proficiency learners showed a significant ACE for the perfective aspect, suggesting a reliance on lexically triggered simulation, while they showed no simulation effect for the progressive aspect due to shallow morphosyntactic processing and L1 transfer. These findings support a proficiency-dependent dual-pathway model of L2 embodiment: advanced learners engage in direct mapping from grammar to simulation, whereas low-proficiency learners rely on an indirect, lexically mediated route. In summary, our findings demonstrate that the embodiment of grammatical meaning in L2 acquisition is not automatic, but is developmentally modulated, evolving from a lexically dependent to grammar-dependent simulation as proficiency increases. Furthermore, these results call for future research to explore the pedagogical applications of grammar-focused embodied instruction and to examine this dual-pathway model across other linguistic structures and L2 populations.

## 1. Introduction

A core claim of Embodied Cognition Theory is that knowledge is grounded in sensorimotor mechanisms (Norman & Peleg, 2022). Accordingly, language comprehension involves unconsciously simulating the sensory–motor experiences described by the language (Barsalou, 1999). Rather than being a purely abstract, amodal process, language processing is believed to recruit the same neural systems used for perception, action and emotion.

Strong behavioral evidence for this simulation account comes from the Action–Sentence Compatibility Effect (ACE) (Glenberg & Kaschak, 2002), which has become a paradigmatic method for investigating sensorimotor simulation in language comprehension (e.g., Winter et al., 2022; Zwaan, 2021). The ACE is found when the direction of action described in the sentence read by the participants is consistent with the direction of the arm movement, the arm movement response is promoted and the reaction time is shortened; otherwise, responses are slower due to motor interference (Cai et al., 2025). In a typical paradigm, participants judge the semantic plausibility of sentences describing transfer actions. To make the task engaging and concrete, the sentences often depict scenarios involving the sharing or withholding of objects (e.g., “You offer the last piece of cake to your friend” vs. “You keep the last piece of cake for yourself”) or the transfer of personal items (e.g., “The student handed the essay to the teacher”). Participants indicate their judgment by making a physical response toward or away from their body. The robust finding, known as ACE, is that responses are significantly faster when the response direction matches the action direction described in the sentence. For instance, responding “away” is faster for the sentence about offering the cake, as if the reader is mentally simulating the act of giving. This interaction between sentence meaning and motor response provides compelling evidence that language comprehension involves mentally simulating the described events.

This paradigm raises a crucial question about the linguistic variables that influence the strength of mental simulation. The grammatical aspect has emerged as a pivotal modulator. Distinct from tense, which locates an event in time (e.g., past, present), the grammatical aspect characterizes the internal temporal contour of an event (Comrie, 1976; Dowty, 1977). The progressive aspect (e.g., is walking, is reading a book) portrays an event from an internal viewpoint, highlighting its ongoing, dynamic progression without regard to the endpoint. The perfective aspect (e.g., has walked, has read a book), instead, offers an external viewpoint, presenting the event as a complete, bounded unit with a focus on its culmination (Becker et al., 2013; Madden & Zwaan, 2003).

By shaping how we conceptualize the temporal flow of an event, aspect is thought to directly guide mental simulation. The progressive, by focusing on the action-in-progress, should potentiate a richer sensorimotor simulation of the action itself, whereas the perfective, by focusing on the end-state, should attenuate such online simulation (Matlock et al., 2007; Anderson et al., 2010; Ferretti et al., 2007). Compelling support for this view comes not only from reaction-time paradigms like the ACE but also from studies on co-speech gestures. For instance, the durative meaning of the progressive aspect is mapped onto metaphoric container gestures, and gestures accompanying progressive utterances are typically longer lasting and more complex than those with non-progressive forms (Matsumoto & Dobs, 2017; Parrill et al., 2013). In fact, research on native English speakers has revealed a robust ACE for progressives but a weakened or absent ACE for perfectives (Bergen & Wheeler, 2010), suggesting that grammatical aspect guides the construal of events during simulation.

While the modulation of ACE by grammatical aspect is well-established in native (L1) processing, it is unclear how this is manifested in second-language (L2) learners. The theoretical link between grammatical aspect and the ACE lies in how aspect modulates the content and timing of mental simulation. The ACE is predicated on the real-time engagement of the motor system during language comprehension. Grammatical aspect directly governs this engagement by shifting the listener’s mental perspective on an action. The progressive aspect, by directing attention to the ongoing action, fosters a dynamic and kinematically detailed motor simulation that is highly susceptible to interference or facilitation from congruent physical movements. This results in a strong ACE. In contrast, the perfective aspect, by directing attention to the completion of an event, promotes a simulation of the end-state or resultant goal, rather than the specific motor processes to achieve it. This decouples the comprehension process from the fine-grained motor system, thereby attenuating or eliminating the ACE.

Given the substantial cross-linguistic differences between English and Mandarin Chinese, this gap is particularly important. Chinese primarily use lexical particles to indicate grammatical aspect, notably “在”[zài] for the progressive and “了”[le] for the perfective. These particles have distinct grammaticalization pathways. Critically, it is hypothesized that the progressive marker “在”[zài] grammaticalized from a spatial meaning (i.e., “to be at”; Ruan & Wang, 2015; W. Wang, 2013). For Chinese learners of English, this basic divergence in the conceptual underpinnings of aspect—especially for the progressive—may pose a major learning challenge, making L2 proficiency an important consideration.

Notably, it has been demonstrated that embodied semantic representation is significantly influenced by L2 proficiency (Ni, 2023; Ahlberg et al., 2018; Birba et al., 2020). However, the evidence is inconclusive regarding the modulation of L2 proficiency on the embodied representations in L2 processing. Several studies have proposed that L2 proficiency modulates L2 embodiment effects (e.g., Q. Wang, 2016; Birba et al., 2020; Gu et al., 2021; Tang et al., 2023). For example, Birba et al. (2020) investigated how embodied semantic systems interacted with L2 proficiency for Spanish–English bilinguals. Participants read action and neutral texts naturally in both languages with their neural activities being tracked by EEG. Results showed higher L2 proficiency correlated with enhanced EEG functional motor-related connectivity during L2 action text comprehension. Similarly, L2 proficiency constrains the ability to represent vertical spatial metaphors related to power words such as “up” and “down” in a second language (Q. Wang, 2016). Nevertheless, according to studies by Chen et al. (2020), Kogan et al. (2020), X. Zhang et al. (2020), L2 proficiency appears to have little effect on the embodied representations in L2 processing. In particular, X. Zhang et al. (2020) examined embodied semantics in L2 processing in an fMRI study. Researchers found that Chinese–English bilinguals, regardless of proficiency level, primarily engaged the semantic systems during the judging of the semantic relatedness of English words, unlike native speakers whose sensorimotor systems were connected with semantic system.

Given these conflicting findings, a critical question arises concerning the predictive power of a major theoretical framework: does the widely accepted neural convergence hypothesis (Green, 2003) hold true for the domain of embodied cognition? This hypothesis states that L2 processing becomes more native-like with increasing proficiency. It follows that high-proficiency learners should develop a different representation from low-proficiency ones, potentially including embodied simulations, due to prolonged exposure and practice. Critical evidence for this progression comes from neurocognitive studies using Event-Related Potentials (ERPs). Specifically, research has leveraged the P600 component as an index of processing efficiency. P600 component is a robust electrophysiological marker of syntactic processing difficulty, including structural reanalysis, integration costs, and the violation of syntactic rules (e.g., Osterhout & Holcomb, 1994). Findings of an attenuated P600 in advanced learners in processing English tense-aspect morphology are thus interpreted as direct evidence of more automatic processing of English tense-aspect morphology (Geng & Yang, 2016; H. Zhang et al., 2017). However, while this compelling evidence for neural convergence is confined to the syntactic domain, it remains unknown whether this proficiency-driven convergence holds true for the embodied simulation of grammatical aspect concepts—that is, for online sensorimotor modulation.

The current work uses the ACE paradigm to fill this gap and elucidate the proficiency-dependent cognitive mechanisms underlying aspectual simulation. However, a clear research gap remains. First, at the behavioral level, it is unknown whether and how L2 learners demonstrate the ACE modulated by grammatical aspect. Second, at a theoretical level, it is unclear whether the Neural Convergence Hypothesis extends from syntactic processing to the domain of embodied semantic simulation. Finally, given the fundamental differences in how aspect is marked in English and Mandarin, it is critical to test whether this theoretical framework holds for this specific language pair. The present study, therefore, has a clear twofold purpose: (1) to empirically investigate how L2 proficiency modulates the embodied simulation of grammatical aspect in Chinese learners of English, using the ACE paradigm; and (2) to test the boundaries of the Neural Convergence Hypothesis in the domain of embodied cognition. Theoretically, this research addresses a critical gap by determining whether proficiency-driven convergence extends from syntactic processing to embodied simulation. Practically, elucidating how learners internalize grammatical concepts like aspect can inform language pedagogy, highlighting the need for instruction that fosters not only structural knowledge but also the corresponding conceptual and embodied representations.

Our predictions are grounded in two key theoretical perspectives. First, the Neural Convergence Hypothesis (Green, 2003) leads us to anticipate that higher-proficiency learners will exhibit embodied simulation patterns that more closely resemble those of native speakers. Second, drawing on theories of L1 conceptual transfer (Jarvis & Paavlenko, 2008) and the critical role of developing strong form–meaning mappings (DeKeyser, 2007), we predict that lower-proficiency learners will show substantial influence from their L1 system. This perspective is strongly supported by research on event categorization. Vanek (2019) demonstrated that even advanced Chinese learners of L2 English retained a persistent L1-tuned, result-oriented bias when categorizing events. Critically, this L1 bias could be rapidly overridden when learners explicitly engaged their L2 through verbalization, which strengthened attentional biases toward the action-oriented categories of English. This evidence highlights that the influence of L1 conceptual patterns and their modulation by L2 engagement are central to understanding L2 semantic processing, directly informing our predictions regarding grammatical aspect.

Our predictions lead to the following specific hypotheses: (1) A significant interaction will exist between grammatical aspect (progressive vs. perfective) and L2 proficiency on the magnitude of the ACE. (2) A specific cross-over interaction is anticipated: advanced learners would show a selective simulation mechanism, with embodied simulation being more robust for progressive than perfective aspect due to strengthened L2 form-concept mappings, as demonstrated by an ACE for progressive but not perfective sentences. This pattern is predicted by the Neural Convergence Hypothesis, as it would mirror the native-like, aspect-sensitive simulation observed in L1 speakers (Bergen & Wheeler, 2010). In contrast, lower-proficiency learners would demonstrate a different pattern, exhibiting a decreased or reversed modulation of simulation by grammatical aspect, potentially showing no ACE or a reversed effect, due to weaker form–meaning associations and interference from L1 conceptual transfer. This is consistent with models of second language acquisition which posit that at early stages, learners rely more heavily on L1 conceptual representations (Jarvis & Paavlenko, 2008), as robustly demonstrated in the domain of event categorization by Vanek (2019), and have less automatic access to L2-specific grammatical distinctions (e.g., Jiang, 2000). By examining this interplay, this study will offer critical evidence on how proficiency shapes the embodied mechanisms of grammatical aspect processing in L2, highlighting the development of form–meaning connections in bilingual cognition.

In summary, this study is designed to uncover the proficiency-dependent cognitive mechanisms through which L2 learners embody grammatical meaning. To test these hypotheses, the following experiment employed the ACE paradigm with Chinese learners of English at varying proficiency levels, as detailed in the Section 2.

## 2. Methods

### 2.1. Participants

The experiment initially recruited 50 Chinese graduate students majoring in English and 50 non-English-major sophomores. All 100 participants completed the Oxford Quick Placement Test (OQPT; Oxford University Press, 2001). Based on their test scores, 32 advanced English learners (scoring above 48 points; 24 females, 8 males) and 32 lower intermediate learners (scoring 24–30 points, 22 females, 10 males) were selected for the main study. It is critical to note that this final group assignment was determined exclusively by participants’ performance on the standardized proficiency test, not by their academic status (i.e., being a graduate student or a sophomore). The recruitment from different academic stages was a sampling strategy to efficiently access populations likely to exhibit divergent proficiency levels. To maximize the difference in proficiency between the two groups, those with mid-range scores were eliminated. None of them had studied overseas, according to the data we gathered on their age, years of English study, and overseas educational experiences. All participants had normal or corrected-to-normal vision and were all right-handed. After being fully informed about the experimental procedures and data collection, all participants provided written informed consent. Each participant received CNY 30 as compensation. Key characteristics of the participants are summarized in Table 1.

### 2.2. Stimuli

Sentence pairs were adapted (with some modifications) from stimuli used by Bergen and Wheeler (2010) and N. Liu and Bergen (2016). The stimuli consisted of seventy sentences in both the perfective and progressive aspects. Critically, these aspects impose different perspectives on the described actions: the perfective aspect (e.g., “He has closed the drawer”) presents the action as a bounded, completed event, whereas the progressive aspect (e.g., “He is closing the drawer”) presents it as an ongoing, unbounded process. This grammatical manipulation allows us to test whether the simulation of motor actions (i.e., the ACE) is sensitive to the temporal perspective encoded by aspect. These 70 sentences were then evaluated in a norming study to select those that most clearly encoded motion direction (toward or away from the body).

In the norming study, 49 Chinese EFL learners (freshmen) were instructed to judge the direction of the hand movement described in each sentence, selecting from three options: *toward*, *away* or *neither*. This cohort was distinct from the participants in the main experiment. We intentionally used freshmen as raters for two reasons: first, the norming task required a basic, perceptual judgment of action direction that we expected to be consistent across proficiency levels; second, this ensured that the main experimental participants had no prior exposure to the stimuli.

Response frequencies were coded. The criteria for including a sentence in the final stimulus set were as follows: (1) High Agreement: At least 50% of participants agreed on a single direction (either “toward” or “away”). (2) Low Ambiguity: Fewer than 25% of responses indicated the opposite direction. Sentences that failed to meet either of these inclusion criteria were excluded. This selection procedure resulted in a final set of 45 clear directional sentences: 22 sentences encoding a “toward” direction and 23 sentences encoding an “away” direction.

Additionally, we assessed the following variables using 7-point Likert scales (1 = lowest, 7 = highest): the relative embodiment of verbs (Sidhu et al., 2014), the familiarity of the action objects, and their imageability. These ratings are summarized in Table 2. Examples of stimuli are shown in Table 3.

### 2.3. Design and Procedure

A total of 45 experimental sentence pairs were created: 22 encoded a “toward” direction (i.e., movement oriented toward the participants’ body), and 23 encoded an “away” direction (i.e., movement oriented away from the participants’ body). This final distribution was a direct result of applying the pre-defined selection criteria during the norming study, which prioritized directional clarity over forced numerical balance. Each pair was presented in both perfective and progressive aspects, resulting in 90 individual sentences. These sentences were evenly divided into two lists, with just one aspectual version of each sentence appearing in a given list.

**Figure 1:**
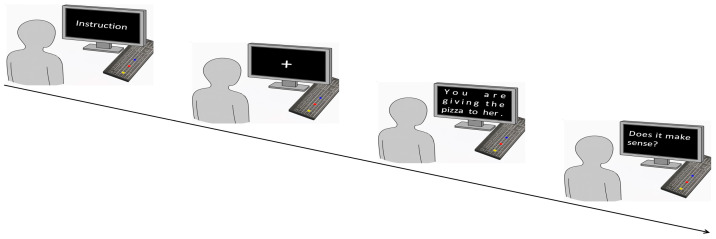
The trial procedure.

A total of 58 filler sentences with semantically anomalous verb–object pairings were added to each list. Participants were informed that the study examined the speed and accuracy of plausibility judgments. They were instructed to perform a motor response (e.g., a forward movement for “plausible” and a backward movement for “implausible”) to indicate whether the sentence described a plausible situation. This cover story was used to ensure that motor reactions to the critical sentences were natural and spontaneous, rather than being influenced by participants’ hypotheses about the relationship between action and language. A schematic overview of the experimental design is presented in Figure 1.

Four experimental versions were produced by counterbalancing these sentences across the two Response Direction conditions (“Yes-is-far” and “Yes-is-close”). In a cross-over design, each participant completed two of the four versions, with a two-week interval between sessions to minimize practice effects. This design was selected to control for item-specific effects (by exposing each participant to a larger portion of the critical stimuli across both aspectual forms) and to minimize carryover effects.

A standard keyboard was used for response collection due to its standardization, high temporal precision for measuring reaction times, and suitability for required directional motor responses. To implement the directional response (toward/away from the body), the keyboard was rotated 90° counter-clockwise and aligned with the participant’s sagittal axis. This setup created an intuitive mapping: the key closer to the torso represented a “toward” movement, and the key farther from the torso represented an “away” movement, thereby physically instantiating the compatibility effect under investigation. Each trial began with a fixation cross. Participants then pressed and held a start button (H key) to display a sentence. After reading, they released the start button and pressed either “Yes” (A key) or “No” (apostrophe key) button to indicate their sentence plausibility judgment. A training session of 8 trials preceded the experiment.

Based on previous studies (e.g., Glenberg & Kaschak, 2002; Borreggine & Kaschak, 2006), the Action–Sentence Compatibility effect (ACE) for sentences with second-person arguments (like all our stimuli) is typically observed in the total response time (i.e., the interval from sentence onset to response button press). Consequently, total response time was adopted as the dependent measure. The experiment had three independent variables: (1) consistency between the direction of motion described by a sentence and the direction of the response arm movement (consistent vs. inconsistent), (2) grammatical aspect (progressive vs. perfective), and (3) English proficiency (advanced vs. lower-proficiency learners).

### 2.4. Results

For the analysis of sentence plausibility judgment task, data from participants with an accuracy rate below 70% were excluded. Individual trials were also removed if the response was incorrect or if the response latency exceeded 2.5 standard deviations from the participant’s mean latency. No participants were excluded from the advanced group based on accuracy, whereas 9 participants were excluded from the low-proficiency group due to low accuracy. Following these exclusions, 13.4% of trials from the advanced group and 24% from the low-proficiency group were removed as incorrect responses.

#### 2.4.1. Accuracy Results

The sentence plausibility judgment task served two purposes. First, it acted as a cover story to guarantee the authenticity of the reading comprehension and reaction time data by requiring participants to assess sentence plausibility and indicate their judgment through their motor responses. Second, accuracy itself is a critical cognitive measure that reflects processing efficiency, cognitive load, and task difficulty. For instance, fast reaction times coupled with low accuracy may indicate that the task was challenging, leading to a speed–accuracy trade-off.

Table 4 displays the accuracy rates for the two participants groups. As shown in the table, advanced learners demonstrated consistently high accuracy (above 87%) across all conditions, with minimal variation between consistent and inconsistent trails or between progressive and perfective aspects. In contrast, low-proficiency learners showed lower overall accuracy (ranging from 76.09% to 81.75%) and greater variability across conditions. Notably, their performance was highest in the perfective aspect with consistent trials (81.75%) and lowest in the progressive aspect with consistent trials (76.09%).

**Figure 2:**
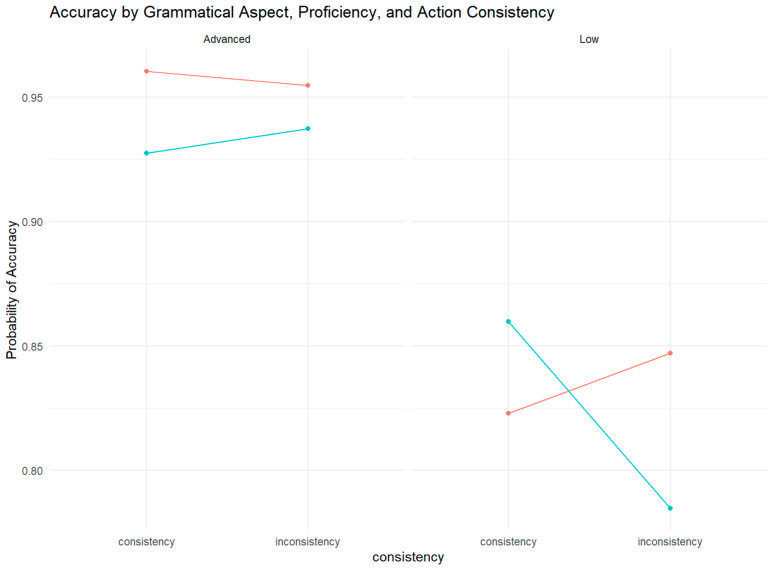
Accuracy by grammatical aspect, language proficiency and sentence–response–direction consistency. The red line represents the progressive aspect, and the blue line represents the perfective aspect.

To statistically evaluate these patterns, we fitted a generalized linear mixed-effects model (GLMM) to the binary accuracy data using the glmer function from the lme4 package (version 1.1-35.1; Bates et al., 2015) in R (version 4.4.2; R Core Team, 2024). The model included Aspect, Consistency, Proficiency, and their interactions as fixed effects, with by-participants and by-item random intercepts. The reference levels were set to “progressive” for aspect, “consistent” for consistency, and “advanced” for proficiency. The results of this model are visually summarized in Figure 2 and detailed below.

The model results showed that grammatical aspect (χ^2^(1) = 4.15, *p* = 0.04) and language proficiency (χ^2^(1) = 39.14, *p* < 0.001) had a significant main effect. The effect of consistency between sentence direction and response direction was not significant(χ^2^(1) = 2.45, *p* = 0.11). Importantly, we found a significant two-way interaction between grammatical aspect and language proficiency (χ^2^(1) = 5.17, *p* = 0.02), as well as a significant three-way interaction between grammatical aspect, consistency between sentence direction and response direction, and language proficiency (χ^2^(1) = 8.2, *p* = 0.004).

The main effects were not interpreted separately because of the three-way interaction. To decompose this interaction, we performed a simple effects analysis. According to these analyses, the interaction between grammatical aspect and consistency was significant among low-proficiency learners (β = −0.7, SE = 0.25, z = −2.8, *p* = 0.005), but not for advanced learners (β = 0.3, SE = 0.3, z = −1.01, *p* = 0.31) (as shown in Figure 1). Specifically, for low-proficiency learners, consistency significantly affected accuracy in the perfective aspect condition (β = 0.52, SE = 0.16, z = 3.26, *p* = 0.001) but not in the progressive aspect condition (*p* = 0.35). For advanced learners, consistency did not significantly affect accuracy in either the progressive (*p* = 0.53; or perfective (*p* = 0.41) aspect conditions.

#### 2.4.2. Response Time Results

To analyze response times (RTs), we fitted a generalized linear mixed model with a log link function and a Gamma distribution. This distribution was chosen because the residuals from an initial normal linear mixed model were right-skewed (skewness = 1.47) and heteroscedastic, and RT data are strictly positive and typically follow a Gamma distribution. The model included grammatical aspect, consistency, language proficiency, and their full interactions as fixed effects. Random intercepts for participants and sentences were included to account for individual and item variability. The model was fitted using the glmer function from the lme4 package in R. Model diagnostics confirmed a non-singular fit (isSingular(gamma_model) = FALSE). The model formula is provided in the Supplementary Materials.

Table 5 displays the results of the model analysis. Parameter estimates (β) represent changes on the model’s log-transformed scale. For the reference conditions (progressive aspect, consistent, advanced proficiency), the intercept represents the predicted mean log (RT).

To visualize and interpret the complex three-way interaction among grammatical aspect, consistency, and proficiency, we present an interaction surface plot in Figure 2. This figure illustrates the estimated log-transformed reaction times across the different experimental conditions.

**Figure 3:**
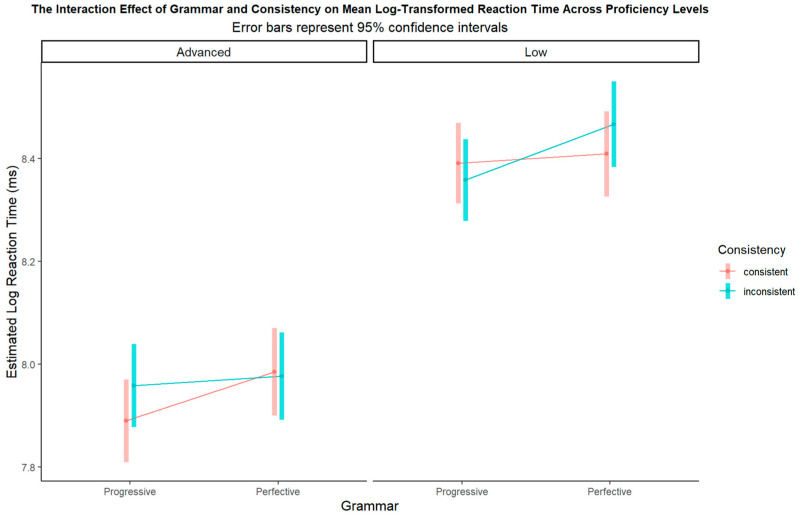
Three-way interaction surface plot between grammatical aspects, consistency, and language proficiency.

The results showed a significant main effect of language proficiency (β = 0.52, SE = 0.06, z = 9.06, *p* < 0.001), indicating that the low-proficiency group responded significantly slower overall than the advanced group. More importantly, a significant three-way interaction was observed (estimate = 0.17, SE = 0.04, z = 4.77, *p* < 0.001). As visualized in Figure 3, this interaction reveals distinct patterns for the two proficiency groups.

We conducted post hoc simple effects analyses to decompose this interaction (as illustrated in Figure 3). For advanced learners, pairwise comparisons revealed two statistically significant differences. First, for progressive sentences, reaction times were significantly faster in the consistent condition than in the inconsistent condition (estimate = −0.065, SE = 0.026, z = −2.54, *p* = 0.01). Second, in the consistent condition, progressive sentences exhibited significantly faster reaction times than perfective sentences (estimate = −0.11, SE = 0.05, z = −2.15, *p* = 0.03). Importantly, there was no difference between consistent and inconsistent conditions for perfective sentences (estimate = 0.009, SE = 0.02, z = 0.51, *p* = 0.61); no other comparisons were significant (all *p* > 0.05).

The pattern for the low-proficiency group was different. We found a significant effect of consistency for perfective sentences: response times were significantly faster in the consistent condition than in the inconsistent condition (estimate = −0.055, SE = 0.018, z = −3.018, *p* = 0.003). Additionally, in the inconsistent context, responses to progressive sentences were significantly faster than those to perfective sentences (estimate = −0.12, SE = 0.05, z = −2.45, *p* = 0.01). No other comparisons reached significance. This pattern suggests that low-proficiency learners are particularly attentive to contextual cues, especially when processing more difficult or unconventional grammatical structures like the perfective aspect. Their representation of this aspect might still be less entrenched and heavily reliant on supportive contextual information, as seen by the higher processing cost for perfective sentences in inconsistent contexts. Progressive sentences may be somewhat more resistant to interference in challenging (inconsistent) conditions because of their higher frequency or earlier acquisition.

## 3. Discussion

This study investigated the embodied simulation of grammatical aspect in L2 learners by examining two key questions: (1) whether grammatical aspect modulates embodied simulation during L2 sentence comprehension, and if any, in what manner; and (2) whether L2 proficiency modulates the way grammatical aspect influences embodied simulation. Our results provide clear answers: grammatical aspect does indeed modulate embodied simulation (as measured by the Action–Sentence Compatibility Effect), but this modulation is profoundly and qualitatively shaped by language proficiency, as evidenced by a significant three-way interaction. This proficiency-dependent pattern reveals unique simulation processes at different learning stages and calls into question oversimplifies conceptions of L2 embodiment.

### 3.1. The Modulation of Embodied Simulation by Grammatical Aspect in Advanced Learners

For advanced learners, the key finding was that grammatical aspect powerfully influenced embodied simulation, as measured by the presence or absence of the Action–Sentence Compatibility Effect (ACE). Embodied simulation was robustly engaged for the progressive aspect, as shown by significantly faster responses in consistent trials compared to inconsistent trials (*p* = 0.022). The perfective sentences, on the other hand, did not cause any such simulation of the directional action: there was no significant difference in response times between consistent and inconsistent conditions (*p* = 0.61).

This aspect-specific pattern aligns with simulation-based theories of language comprehension (Bergen & Wheeler, 2010), which posit that grammatical aspect provides high-level constraints (cf. Feldman & Narayanan, 2004) that define the type of the mental simulation. The progressive aspect is thought to trigger a mental simulation of the ongoing activity (e.g., “was walking”), which shares neural resources with the actual motor action required by the task. Under congruent conditions, this overlap facilitates processing, but it causes interference under inconsistent conditions, manifesting as the observed ACE. Conversely, the perfective aspect (e.g., “had walked”) shifts the focus to the endpoint or completion of an event. This type of simulation may not activate the specific motor programs engaged by our ACE paradigm, explaining the absence of a compatibility effect.

These results demonstrate that advanced L2 learners, much like native speakers, engage in intricate sensorimotor simulation that is highly sensitive to grammatical cues. This finding contributes to the growing body of research on embodiment effects in L2 processing (e.g., Q. Wang, 2016; Y. Zhang & Zhang, 2015), and crucially extends it from the lexical level to the domain of grammar. As Bergen and Wheeler (2010) suggest, the influence of grammatical aspect implies that it functions as a “simulation specification”—a higher-order constraint that parameterizes how learners activate perceptual and motor systems to interpret sentences.

### 3.2. The Reversal of Simulation Patterns in Low-Proficiency Learners

The pattern of embodied simulation for low-proficiency learners differed qualitatively from that of advanced learners, confirming that proficiency fundamentally modulates the way grammatical aspect affects simulation. Simple effects analyses revealed a striking reversal: perfective sentences showed a significant ACE, with faster responses in consistent condition than in inconsistent conditions (*p* = 0.003), suggesting that the perfective aspect successfully triggered simulation. In contrast, no ACE was found for progressive sentences (*p* > 0.05), indicating that action simulation was not engaged for this aspect. Furthermore, responses to progressive sentences were significantly faster than those to perfective ones in the inconsistent condition, which poses higher cognitive demands (*p* = 0.01).

#### 3.2.1. A Shallow Processing Account for the Progressive Aspect

We suggest that this reversal pattern stems from a qualitative difference in how low-proficiency learners process each aspect. As evidenced by Geng and Yang (2016), the progressive aspect is associated with a lower syntactic processing cost (smaller P600 amplitude) than the perfective aspect, a pattern which suggests that learners process the “Be + V-ing” construction as a holistic formal unit. However, this formal recognition is not accompanied by a robust, automatic retrieval of the form–meaning connection to its aspectual semantics of ongoing activity. As a result, it fails to trigger the action simulation necessary to produce an ACE. This notion of shallow, conflict-free processing is further supported by the faster reactions to progressive sentences in the demanding inconsistent condition.

#### 3.2.2. Lexically Driven Simulation in the Perfective Aspect

In contrast, learners have trouble integrating morphosyntactic elements for the perfective aspect (“have + V-en”), as indicated by a higher P600 (Geng & Yang, 2016). As a result, learners might be forced to concentrate on the main verb and use a discrete, lexical-processing approach. The concrete, directional semantics of the verb (e.g., “push”, “pull”) can then directly trigger an action simulation, thereby yielding the observed ACE. This account explains the key findings: the ACE emerges for the perfective aspect due to verb-driven simulation, but is absent for the progressive aspect due to lack of simulation. It also clarifies why response times were faster for progressive sentences under inconsistent condition: shallow formal analysis proceeds efficiently, whereas deep simulation encounters costly conflict. This is in line with the recognized developmental sequence in second-language learning, where bare verbs are acquired first, followed by V-ing forms, with complex periphrastic compounds like “have + V-en” acquired last (Hawkins, 2001; Yu, 2008). The later acquisition of the perfective aspect may explain why low-proficiency learners struggled with its morphosyntactic integration and resort to lexical-driven processing.

#### 3.2.3. The Role of L1 Transfer and Typological Differences

Beyond these developmental processes, cross-linguistic transfer from the native language could be an extra source of interference. The Chinese and English aspectual systems differ fundamentally in typology. The progressive aspect of English is realized through a morphosyntactic construction requiring both an inflected form of the auxiliary verb BE and the morphological inflection of the main verb into the -ing form. This highly grammaticalized structure encodes primarily temporal information, emphasizing the ongoing and incomplete nature of an action. However, Chinese progressive aspect markers like “在 (zài)” and “着 (zhe)” are not inflectional and, being grammaticalized from originally spatial verbs, often retain semantic connotations of spatial continuity (Ruan & Wang, 2015). Therefore, Chinese tends to express aspectual meaning through lexical and pragmatic ways within primarily spatial–conceptual frameworks (Zhao & Wang, 2017). This typological disparity may lead Chinese learners of English to adopt L1-based processing strategies. This is supported by Ma and Du’s (2020) findings that low-proficiency Chinese EFL learners did not use native-like ambiguity resolution mechanisms that rely on aspectual cues, revealing that they were less sensitive to grammatical aspects when processing sentences with progressive morphology (e.g., -ing forms) online. The absence of an ACE for the progressive aspect can be explained by this lack of sensitivity, which renders the compatibility between sentence and response direction irrelevant. Conversely, the observed ACE for the perfective aspect may result from learners relying more on parsing discrete morphological cues and lexical verb semantics, which can directly trigger concrete action simulations.

#### 3.2.4. Complementary Evidence from Response Accuracy

Crucially, the analysis of response accuracy provides complementary evidence that further elucidates the nature of this lexically driven simulation. For low-proficiency learners, the consistency between sentence direction and response direction not only influenced the speed of their responses to perfective sentences (as seen in the RT-based ACE) but also significantly affected the accuracy of their semantic judgment (β = 0.52, SE = 0.16, z = 3.26, *p* = 0.001). This pattern—where consistent trials yield higher accuracy than inconsistent trials—is a classic signature of high cognitive load. It indicates that when simulation is engaged via the lexical verb in the perfective aspect, the conflict in inconsistent conditions is so demanding that it compromises the final judgment, leading to more errors. This finding directly supports our interpretation that low-proficiency learners employ an effortful and strategic processing route for the perfective aspect, where simulation is not automatic but comes at a cost to both processing efficiency and outcome reliability.

In stark contrast, for the progressive aspect, where no ACE was observed in RTs, accuracy was also unaffected by consistency (*p* = 0.35). This double dissociation—no effect on either speed or accuracy—robustly confirms the “shallow processing” account. It suggests that the progressive aspect fails to trigger a deep simulation that could interact with the motor system, thereby rendering the compatibility of the sentence direction and arm movement direction irrelevant to the task, both in terms of processing speed and final judgment.

In summary, we contend that the proficiency-dependent reversal of the ACE is caused by a fundamental difference in processing strategies: a shallow, form-based processing of the progressive aspect that fails to trigger action simulation, versus a lexically driven, analytical processing of the perfective aspect that successfully engages the sensorimotor system.

### 3.3. Theoretical Integration and Implications

This study goes beyond merely documenting the presence or absence of embodied effects in L2 processing by demonstrating the proficiency-dependent reversal of the ACE. Rather, it reveals a deeper principle: the pathway and efficiency of embodied simulation are dynamically modulated by language proficiency. Our findings challenge the assumption of a universal, direct link between grammar and simulation. Therefore, we propose a dual-pathway model of L2 embodiment, in which the engagement of either pathway is conditioned by developmental readiness and L1-conceptual filters.

This reversal pattern compels a crucial theoretical refinement. The seminal monolingual framework (Bergen & Wheeler, 2010) posits a direct mapping wherein grammatical aspects automatically specify distinct simulation formats (process vs. endpoint). Our data suggest that for L2 learners, this direct grammar → simulation link is not a starting point but rather a developmental accomplishment. For low-proficiency learners, the process is asymmetrical and indirect: grammar → analytical strategy → simulation. Through an alternative, lexically driven route, the perfective aspect, affords a pathway to simulation via its lexical verb. In contrast, the progressive aspect, with its complex morphosyntactic demands, leads to shallow processing that fails to trigger simulation.

The dissociation between reaction time and accuracy patterns across proficiency levels offers a clear behavioral marker of this transition. For low-proficiency learners, the consistency effect manifested in both RT and accuracy for the perfective aspect, underscoring the costliness and strategic nature of their simulation. For advanced learners, however, the effect was isolated to RT (specifically for the progressive aspect) without compromising accuracy. This pattern is indicative of emerging automaticity: the simulation process, while not yet entirely cost-free in terms of speed, has become robust enough to ensure error-free final outcomes. Thus, the progression towards the “direct-link paradigm” is not only reflected in what is simulated (as shown by the reversal of the ACE) but also in how efficiently that simulation is executed, with accuracy being the first to stabilize at native-like levels.

Our data provide behavioral support for Ni’s (2023) integrated model, which positions proficiency as a central moderator governing the interaction between linguistic representations and the simulation system. Specifically, low L2 proficiency impedes direct simulation and increases dependence on L1-mediated analytical processing. The reversal of the ACE is a stark behavioral indicator of this underlying cognitive configuration. Only at advanced proficiency levels does the system reconfigure to allow for a more direct grammar–simulation pathway, minimizing the mediating influence of the L1.

Ultimately, this study argues for a theory of L2 embodied cognition that is fundamentally probabilistic and developmental. Whether and how a grammatical structure will engage the sensorimotor system is not a fixed property of the structure itself, but a function of the learner’s proficiency and the resultant processing strategies it affords. The ACE does not simply “diminish” with lower proficiency; it can fundamentally reconfigure based on the learners’ available linguistic and conceptual resources.

While our behavioral study does not prescribe specific classroom activities, it provides a crucial theoretical foundation for rethinking L2 grammar instruction and outlines clear directions for future applied research. The central implication is that advanced L2 proficiency entails the development of a native-like “embodiment regime”—the automatic capacity to simulate meaning cued by grammar, which goes beyond the mastery of formal rules. This reconceptualization of proficiency from a purely linguistic to an embodied-cognitive level suggests that the ultimate goal of instruction should be to foster these direct form–meaning–simulation mappings.

Our findings raise critical, empirically testable questions for pedagogy: Could instructional techniques that explicitly engage the sensorimotor system (e.g., having learners perform or observe actions while using target grammatical structures) facilitate the development of this direct grammar–simulation link more effectively than traditional, abstract methods? Does the proficiency-dependent shift from lexical to grammatical simulation which we observed predict the ability to use language fluently and intuitively in real-time communication? Future research that translates these cognitive findings into classroom interventions is needed to bridge the gap between the laboratory and the language classroom. Therefore, the primary contribution of this work to pedagogy at present is not a set of teaching rules, but a new theoretical lens and a robust experimental paradigm for rigorously evaluating how embodied learning principles can optimize the acquisition of grammatical meaning.

In conclusion, we propose that the bilingual mind does not merely contain two linguistic systems but potentially two different embodiment regimes. The low-proficiency regime is characterized by effortful, strategically mediated simulation that is highly susceptible to L1 transfer. The high-proficiency regime approximates the monolingual direct-link paradigm. The transition from strategic to automatic embodiment represents a crucial, previously unidentified milestone in advanced L2 acquisition. Our findings, which document this qualitative shift, call for an extension of embodied cognition theory to account for the dynamic and experience-dependent nature of simulation processes in bilinguals.

## 4. Conclusions

This study investigated the embodied simulation of grammatical aspect in L2 sentence comprehension by addressing two central questions: (1) whether grammatical aspect modulates embodied simulation in L2 learners and if so, how, and (2) whether L2 proficiency modulates the way grammatical aspect influences such simulation. Using the ACE as a behavioral index of simulation, mixed-effects models revealed a significant three-way interaction, demonstrating that proficiency qualitatively shapes how grammatical aspect constrains simulation.

The main finding is a proficiency-dependent reversal in simulation patterns. Advanced learners, guided by grammar, showed an ACE for the progressive aspects, which fosters simulation of ongoing action, but not for the perfective aspects, which emphasize event completion. In contrast, low-proficiency learners displayed a reversed pattern: a significant ACE for the perfective aspects, suggesting verb-driven simulation due to difficulties with morphological integration, and no ACE for progressive aspects, indicating shallow, form-based processing that fails to trigger semantic simulation.

Theoretically, these findings support a dual-pathway, developmental model of L2 embodiment wherein simulation mechanisms are dynamically shaped by proficiency. The grammar–simulation link in L2 learners is not inherently direct. Instead, this link is strengthened through the development of robust form–meaning mappings and reduced L1 transfer at higher proficiency levels.

Pedagogically, our findings underscore the importance of building robust grammatical form–meaning connections through enriched input and practice. Technologies like virtual reality may provide multimodal, immersive environments to support embodied learning and facilitate the shift from effortful, lexically mediated simulation to automatic, grammatical guided simulation.

This study has several limitations that should be acknowledged. First, addressing the methodological limitations of the present study, future work should explore a wider range of verbs, employ longitudinal designs, and utilize more naturalistic tasks to enhance the generalizability and ecological validity of our findings.

More profoundly, our “two embodiment regimes” model points to a fundamental question in bilingual cognition: the potential for bidirectional interaction between L1 and L2 embodied systems. While our model traces the development of L2 processing, it raises the compelling possibility that attaining advanced L2 simulation capabilities may come at the cost of, or even restructure, native-like L1 embodied processing (Vanek, 2019; Baker, 2024; Zingaretti et al., 2025). Directly testing for such L1 restructuring in highly proficient bilinguals constitutes a critical next step. Investigating this interplay is essential for moving beyond static models and toward a truly dynamic understanding of how embodied cognition is shaped by the bilingual experience.

In conclusion, this study demonstrates that the embodied simulation of grammatical aspect in L2 learners is a unitary phenomenon but is fundamentally shaped by proficiency. Higher proficiency enables a more direct, target-like interaction between language and simulation, whereas lower proficiency leads to asymmetrical, compensatory simulation routes. The findings suggest that the bilingual mind may function under different embodiment regimes depending on the stage of learning, with advanced proficiency enabling more direct grammar–simulation links.

## Figures and Tables

**Table 1:** Summary of selected characteristics of the participants.

Group	Number	Age	Years of Learning English (Year)	QPT Points
Advanced	32	24.91 (1.96)	14.21 (1.62)	48.743 (1.82)
Low	32	21.86 (1.99)	11.69 (1.68)	28.171 (2.06)

**Table 2:** Characteristics of verbs and nouns in the sentences.

	Toward Direction	Away Direction
relative embodiment of verbs	4.78 (0.5)	4.81 (0.84)
familiarity of objects	5.76 (0.95)	5.93 (1.004)
imageability of objects	4.63 (0.52)	4.99 (0.55)

Note: Independent-samples *t*-tests revealed no significant differences between the two sentence sets on any of these variables (all *p* > 0.05).

**Table 3:** Examples of stimuli.

Grammatical Aspect	Sentence Direction	Example Sentences	Response Direction Required When Judging the Sensibility of a Sentence
progressive	away	You were closing the drawer.	away
			toward
Progressive	toward	You were opening the drawer.	away
			toward
Perfective	away	You had closed the drawer.	away
			toward
Perfective	toward	You had opened the drawer.	away
			toward
Filler	You were chopping the log with a bread *.		away
			toward

* denotes an illogical filler statement to which the correct response is “NO”.

**Table 4:** Accuracy rate for different participant groups.

Proficiency Group	Grammatical Aspect	Consistency	Accuracy Rate
advanced	progressive	toward–toward, away–away	90.79%
		toward–away, away–toward	90.41%
	perfective	toward–toward, away–away	87.28%
		toward–away, away–toward	89.77%
low	progressive	toward–toward, away–away	76.09%
		toward–away, away–toward	77.93%
	perfective	toward–toward, away–away	81.75%
		toward–away, away–toward	77.42%

Note. Consistency categorizes the match between the sentence’s action direction and the participant’s motor response: Consistent: sentence direction and response direction match (e.g., a “toward” sentence with a “toward” response, or an “away” sentence with an “away” response). Inconsistent: sentence direction and response direction mismatch (e.g., a “toward” sentence with an “away” response, or an “away” sentence with a “toward” response).

**Table 5:** Results of the Gamma GLMM analysis.

**Random Effects**	**Variance (S^2^)**		**Std. Dev.**	
Participants (Intercept)	0.02		0.15	
Sentences (Intercept)	0.01		0.12	
Residual	0.11		0.33	
**Fixed Effects**	**Estimate (E)**	**Std. Error (SE)**	**z**	***p***
(intercept)	7.89	0.04	147.56	<2 × 10^−16^ ***
Aspects: progressive → perfective	0.11	0.05	2.15	0.03 *
Direction: consistency → inconsistency	0.07	0.03	2.54	0.01 *
Proficiency: advanced → low intermediate	0.52	0.06	9.06	<2 × 10^−16^ ***
grammar: consistency	−0.07	0.03	−2.37	0.02 *
grammar: proficiency	−0.08	0.03	−2.52	0.01 **
consistency: proficiency	−0.1	0.03	−4.20	2.62 × 10^−5^ ***
Grammar: consistency: proficiency	0.17	0.04	4.77	1.86 × 10^−6^ ***

Note: * *p* < 0.05, ** *p* < 0.01, *** *p* < 0.001.

## Data Availability

The data that support the findings of this study are not available due to their containing information that could compromise the privacy of the research participants. Derivative data and analysis outputs are available from the corresponding author upon reasonable request.

## Supplementary Materials

The following supporting information can be downloaded at: https://www.mdpi.com/article/10.3390/bs15111581/s1, File S1. Statistical output for the post-hoc tests from the analysis of ACE reaction times across different language proficiency groups.
