# Supplementary material for: The Embodied Simulation of L2 Grammatical Aspect: Proficiency-Dependent Evidence from the Action-Sentence Compatibility Effect
Source: Behav Sci (Basel). 2025 Nov 18;15(11):1581. doi: 10.3390/bs15111581 (PMC12649481; doi:10.3390/bs15111581)
Supplement: Supplementary file 1 [file behavsci-15-01581-s001.zip › behavsci-3919558-supplementary.pdf]

ACE reaction times' analysis between different proficiency group.

```
> emm_3way <- emmeans(gamma_clean2, ~ grammar * consistency | proficiency)
> contrast(emm_3way, "pairwise", by = c("consistency", "proficiency"))
consistency = consistent, proficiency = Advanced:
 contrast estimate     SE  df z.ratio p.value
 IM - PER  -0.1058 0.0493 Inf  -2.147  0.0318

consistency = inconsistent, proficiency = Advanced:
 contrast estimate     SE  df z.ratio p.value
 IM - PER  -0.0318 0.0502 Inf  -0.634  0.5261

consistency = consistent, proficiency = Low:
 contrast estimate     SE  df z.ratio p.value
 IM - PER  -0.0274 0.0485 Inf  -0.565  0.5722

consistency = inconsistent, proficiency = Low:
 contrast estimate     SE  df z.ratio p.value
 IM - PER  -0.1232 0.0502 Inf  -2.453  0.0142
```

```
> emm_consistency_proficiency<-emmeans(gamma_clean2,~grammar*proficiency|consistenc
y)
> contrast(emm_consistency_proficiency,"pairwise",by=c("grammar","proficiency"))
grammar = IM, proficiency = Advanced:
 contrast                  estimate     SE  df z.ratio p.value
 consistent - inconsistent -0.06512 0.0256 Inf  -2.541  0.0111

grammar = PER, proficiency = Advanced:
 contrast                  estimate     SE  df z.ratio p.value
 consistent - inconsistent  0.00888 0.0174 Inf   0.509  0.6105

grammar = IM, proficiency = Low:
 contrast                  estimate     SE  df z.ratio p.value
 consistent - inconsistent  0.04075 0.0264 Inf   1.544  0.1226

grammar = PER, proficiency = Low:
 contrast                  estimate     SE  df z.ratio p.value
 consistent - inconsistent -0.05511 0.0183 Inf  -3.018  0.0025
```
